# Information Optimized Multilayer Network Representation of High Density Electroencephalogram Recordings

**DOI:** 10.3389/fnetp.2021.746118

**Published:** 2021-09-28

**Authors:** Francesc Font-Clos, Benedetta Spelta, Armando D’Agostino, Francesco Donati, Simone Sarasso, Maria Paola Canevini, Stefano Zapperi, Caterina A. M. La Porta

**Affiliations:** ^1^ Center for Complexity and Biosystems, Department of Physics, University of Milan, Milano, Italy; ^2^ Department of Health Sciences, University of Milan, Milano, Italy; ^3^ Department of Mental Health and Addiction, ASST Santi Paolo e Carlo, Milano, Italy; ^4^ Department of Biomedical and Clinical Sciences ‘Luigi Sacco’, Milano, Italy; ^5^ CNR-Consiglio Nazionale delle Ricerche, Istituto di Chimica della Materia Condensata e di Tecnologie per l’Energia, Milano, Italy; ^6^ Center for Complexity and Biosystems, Department of Environmental Science and Policy, University of Milan, Milano, Italy; ^7^ CNR-Consiglio Nazionale delle Ricerche, Istituto di Biofisica, Milano, Italy

**Keywords:** high density electroencephalogram, multilayer networks, bipolar disorder, maximum information, first episode psychosis

## Abstract

High-density electroencephalography (hd-EEG) provides an accessible indirect method to record spatio-temporal brain activity with potential for disease diagnosis and monitoring. Due to their highly multidimensional nature, extracting useful information from hd-EEG recordings is a complex task. Network representations have been shown to provide an intuitive picture of the spatial connectivity underlying an electroencephalogram recording, although some information is lost in the projection. Here, we propose a method to construct multilayer network representations of hd-EEG recordings that maximize their information content and test it on sleep data recorded in individuals with mental health issues. We perform a series of statistical measurements on the multilayer networks obtained from patients and control subjects and detect significant differences between the groups in clustering coefficient, betwenness centrality, average shortest path length and parieto occipital edge presence. In particular, patients with a mood disorder display a increased edge presence in the parieto-occipital region with respect to healthy control subjects, indicating a highly correlated electrical activity in that region of the brain. We also show that multilayer networks at constant edge density perform better, since most network properties are correlated with the edge density itself which can act as a confounding factor. Our results show that it is possible to stratify patients through statistical measurements on a multilayer network representation of hd-EEG recordings. The analysis reveals that individuals with mental health issues display strongly correlated signals in the parieto-occipital region. Our methodology could be useful as a visualization and analysis tool for hd-EEG recordings in a variety of pathological conditions.

## 1 Introduction

Recent developments in neuroscience are giving rise to an increasing amount of data on the functioning of the brain at different scales, from molecular processes at the level of single neurons to macroscopic signals encompassing the whole brain, as in electroencephalogram (EEG) or functional magnetic resonance imaging (fMRI). Despite the trove of accumulating data, disentangling the complexity of brain function is still a largely open issue. A particularly important goal is to develop tools that are able to extract useful information from brain activity measurements on individual subjects in order to identify potential network dysfunction and support diagnosis (Bassett, 2021).

It is becoming increasingly clear that brain activity is strongly interconnected and hierarchically organized, requiring a sophisticated mathematical description to infer its underlying properties from measurements. The emerging field of *network neuroscience* is advocating the use of networks descriptions for a statistical analysis of brain functions at multiple spatio-temporal scales (Bassett and Sporns, 2017). As in many other applications, a network representation can be derived by suitably thresholding the covariance matrix of the signal recorded at different locations (Masuda et al., 2018) with sophisticated metodologies to chose an optimal threshold (De Vico Fallani et al., 2017) or using singular value decomposition of the multidimensional signal (Worsley et al., 2005). A typical feature of many complex networks that appears promising to describe the hierarchical brain organization is the small-world topology involving at the same time small-scale local clusters and long-range connections between distant areas (Bassett and Bullmore, 2006). Networks provide a visual representation of brain connectivity (Rubinov and Sporns, 2010), but extracting robust statistical information from brain network is a challenging task. Measures at the intersection between neuroscience and complexity theory have emerged such as topological data analysis (Phinyomark et al., 2017) or multivariate auto-regressive models (Astolfi et al., 2007).

EEG recordings have attracted a wide interest for many years in the study of brain function due to the relative simplicity in which spatially localized time dependent data can be acquired through non-invasive instrumentation. EEG data are conventionally analyzed by sampling time depended signals into different frequency bands at different locations on the scalp and then looking for specific signatures in each band. For instance, resting state EEG in patients diagnosed with First Episode Psychosis and Bipolar Disorder revealed a general trend of increased delta (0.5–4 Hz) and theta (4–8 Hz) activity, and a decrease in alpha (8–13 Hz) activity (Clementz et al., 1994). Resting state EEG of bipolar patients has also been studied using complex network analysis in Kim et al. (2013), yielding differences from healthy control subjects across several network measures such as clustering coefficient or characteristic path length. More recently, machine learning combined with complex network analysis was used to classify non-epileptic and epileptic EEG signals (Gao et al., 2020). Network analysis was also performed for EEG signals recorded in Alzheimer Disease patients during cognitive tasks and resting state (Das and Puthankattil, 2020), revealing a higher betweenness centrality in patients compared to controls subjects.

Since EEG signals are highly multidimensional, considering their dependence on time, location and frequency band, a projection into a single network may overshadow some essential feature of the system. To overcome this limitation, multilayer netwroks have been recently proposed as a promising tool to study the dynamics of brain activity, reducing the information loss due to the projection into a single network (Muldoon and Bassett, 2016; De Domenico, 2017). A multilayer network can be seen as an interconnected set single-layer networks where each layer represents a particular dimension of the original signal (Aleta and Moreno, 2019; Bianconi, 2018). In the context of EEG we can assign distinct layers to different time windows and/or different frequency bands and assign each electrode to a node in each single-layer network. For example, a time-based multilayer complex network analysis was perfomed on EEG recordings in patients with epilepsy (Leitgeb et al., 2020). The central issues in multilayer network based methods for EEG signal is to find a representation that minimizes information loss and introduce suitable statistical tools to extract readable information from the networks.

In this paper, we propose a multilayer network representation of EEG signals that maximize the information content and apply it to a set of sleep EEG data from patients diagnosed with First Episode Psychosis (FEP) or Bipolar Disorder (BD) and compared with control subjects. We then use a set of network measures and show that it is easier to reliably stratify patients from control subjects when using network representations with constant edge densities.

## 2 Results

### 2.1 Maximization of Total Information Change Over Time

**FIGURE 1:**
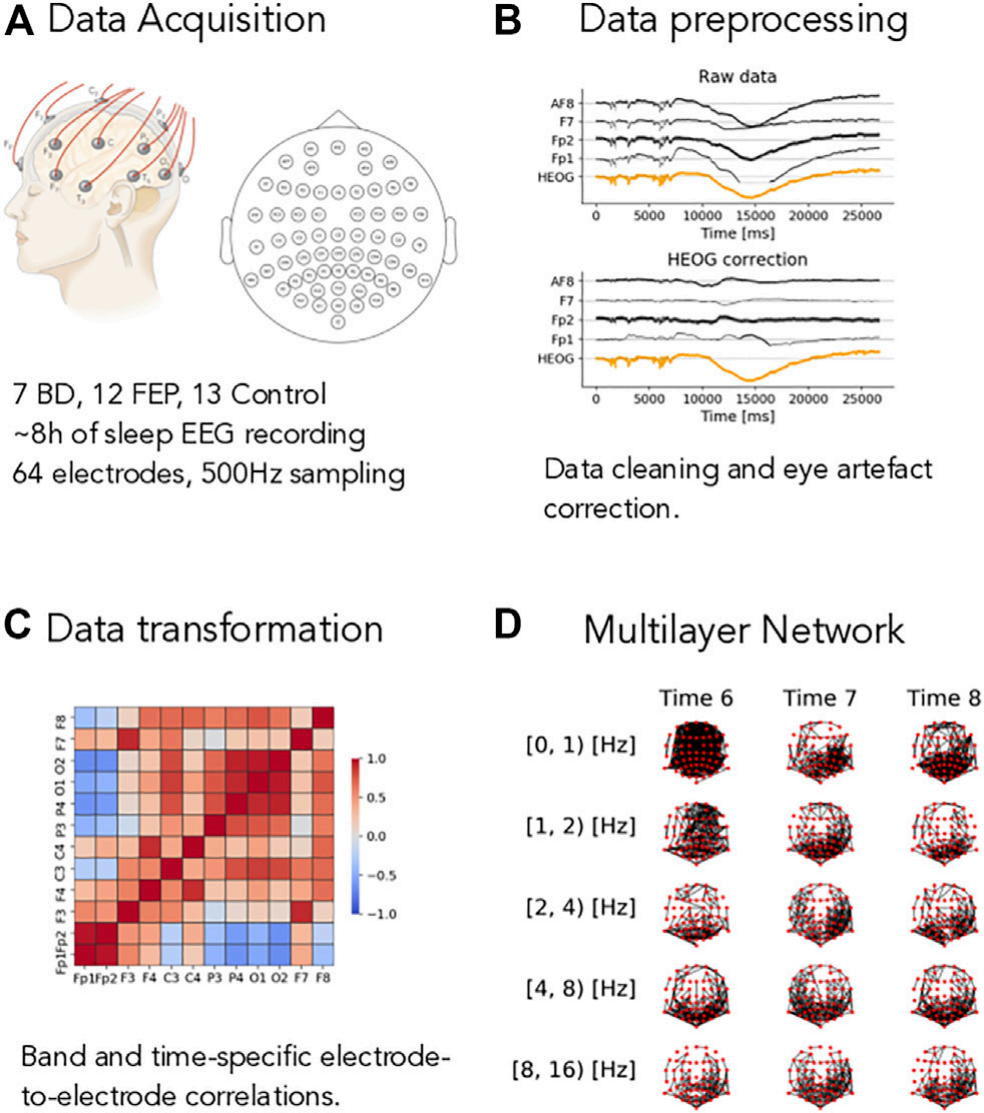
Overview of the method. **(A)** Short description of the dataset, see Methods for complete details. **(B)** Example of the eye-artifact correction method, showing the correlation of the signal from electrodes AF8, F7, Fp1, and Fp2 (black lines) with horizontal eye movements (HEOG, orange line) before (Raw data panel) and after (HEOG correction panel) the eye-artifact correction method. **(C)** Example of a electrode-to-electrode correlation matrix, depicted as a heatmap. Correlation goes from −1 (blue shading) to +1 (red shading). Correlations are both time- and band-specific. **(D)** Example of the resulting band- and time-depended multilayer networks, where nodes represent electrode and edges represent high correlations, see Methods for the details of the different thresholding procedures.

Sleep hd-EEG recordings from 12 FEP, seven BD patients, and 13 control subjects were analyzed, see Methods for details and Figure 1. Raw data are extremely fine-grained: the sampling frequency of 500 Hz during an average of 8 h of sleep, multiplied by the 64 electrodes that comprise the EEG headset yields approximately, 1,000,000,000 measurements per patient. Clearly, these measurements are not all independent of each other, but they encode information that spans several sleep phases and brain regions. Therefore, we aim at finding a satisfactory compromise between compression and information.

To do so, we process the raw sleep EEG records through our pipeline as described in Methods in detail and illustrated in Figure 1. The first step is to remove artifacts from the data. Eye-movement artifacts are well known to influence raw sleep EEG data. To mitigate their impact on our results, we use a fast linear regression model to correct for eye movements, see Methods for details and Figure 1B: in this illustrative figure, the horizontal electro-oculogram potential (HEOG) well correlated with channels AF8, F7, FP2 and FP1 in the top plot. After the correction step (bottom plot in pannel B), this dependence was almost completely eliminated. After splitting the signal into different frequency bands (see Methods for details), we compute time- and band-specific electrode-to-electrode correlations of the form $C_{ij}^{b}\left(t\right)$ , represented as a heatmap in Figure 1C. Finally, we construct time-varying multilayer networks using an innovative strategy that takes into account the whole dataset (and not each time snapshot individually), maximising the total amount of information contained in the time-varying dataset. Figure 1D offers a visual representation of the final output we obtain after processing the raw EEG data: a set of time-varying multilayer networs, where different layers correspond to different frequency bands, network nodes represent electrodes and edges represent high EEG correlations.

**FIGURE 2:**
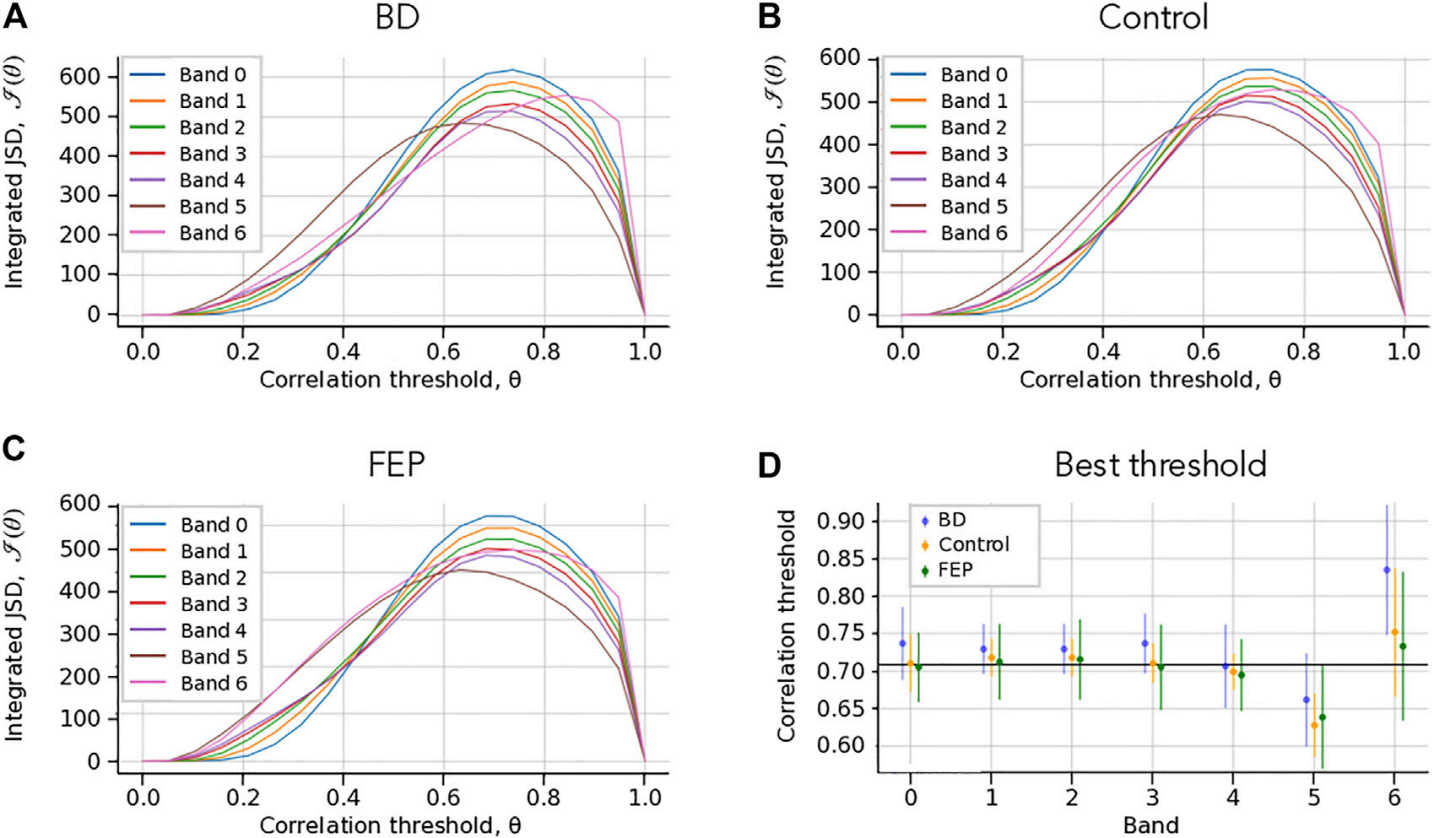
Choice of correlation thresholds. **(A–C)** Check Which Patient. Integrated Jensen-Shannon divergence (JSD) as a function of the correlation threshold *θ*, for each band (colored lines), for BD **(A)**, Control **(B)** and FEP **(C)** patients. The panel shows a consistent maximum of the integrated JSD at around *θ* = 0.7. **(B)** Threshold that maximizes the integrated JSD. The errorbars correspond to the average over different patients. The overall chosen best threshold is marked as a solid horizontal black line, see Methods for details. The panel shows that a single correlation threshold value can accommodate all patient groups and frequency bands.

Networks offer a simplified and effective representation of interactions between nodes, but deciding the correlation threshold beyond which edges are added to the network is a nontrivial subject. In order to make an informed choice, here we introduce the Integrated Jensen-Shannon Divergence (IJSD),

$$I\left(\theta \right)=\overset{\theta }{\underset{t=1}{\sum }}D\left(\rho_{t-1},\rho_{t}\right),$$ (1)

a measure of the total information change over time (Grosse et al., 2002), computed as the sum of the Jensen-Shannon divergence of each epoch with respect to the previous one. Here *ρ*_*t*_ are the density matrices associated to each network in the framework of spectral entropies (Domenico and Biamonte, 2016), see Methods for details. The value of I depends on *θ* in non-trivial ways, but the limit cases are clear: if *θ* is too low (high), all edges are present (absent) at all time steps, so there is no information change over time and thus I=0 for both *θ* = 0 and *θ* = 1. It is only for intermediate values of the correlation threshold *θ* that the sequence of multilayer networks can display richer temporal variations, yielding a higher information change. This can be clearly seen in Figure 2 panels (A, B, C), which show the value of I as a function of *θ* for one BD, one FEP and one control example. As anticipated, I(θ)=0 for both *θ* = 0 and *θ* = 1, with a clear maximum at around *θ* ∼ 0.7 for most frequency bands.

### 2.2 Fixed-Threshold and Fixed-Density Networks

We implement two strategies to choose the optimal correlation threshold θ* from the analysis of the information content quantified by IJSD. In the first approach, we set a global absolute value for the correlation threshold, while in the second approach that value is relative to each network and chosen to maintain a constant edge density, keeping only the interactions with highest absolute correlation. In both cases, the adjacency matrices can be build as

$$A_{ij}^{b}\left(t\right)=\left\{\begin{array}{cc} 1\; & \text{if }|C_{ij}^{b}\left(t\right)|\geq \theta^{*}\\ 0\; & \text{else} \end{array}\right.$$ (2)

that is, we place edges for both large positive and large negative correlations.

The optimal correlation threshold θ* for fixed-threshold networks is computed as the average of the band- and patient-specific optimal values that result from optimizing each case separately,

$$\begin{array}{ll} \theta^{*} & ={\left\langle \theta_{b,p}^{*}\right\rangle }_{b,p} \end{array}$$ (3)

$$\begin{array}{ll} \theta_{b,p}^{*} & ={argmax}_{\theta \in \left[0,1\right]}I_{b,p}\left(\theta \right) \end{array}$$ (4)

where $I_{\theta }\left(b,p\right)$ denotes the IJSD of patient *p* at frequency band *b*. In other words, for each patient *p* we compute a band-specific optimal threshold $\theta_{b,p}^{*}$ . The group averages and variability of these are shown in Figure 2. Taking the average of all $\theta_{b,p}^{*}$ , we reach an overall value of θ* = 0.72, shown as a black solid line in Figure 2. Overall, the figure shows that a single global threshold can reasonably accommodate for the band- and patient-specific optimal values.

The second approach consists in keeping the same fraction of edges in all networks, yielding what we call *fixed-density* networks. The optimal density value in this case is set so that it coincides with the average density of the fixed-threshold networks. This second approach takes into account that different patients, time point or bands might have different intrinsic correlation levels, and presents additional advantages from the network analysis point of view.

### 2.3 Network Edge Presence Shows Differences Between Groups

We investigate the group differences between BD and control patients, as well as between FEP and control patients. To do so, we need to condense the information contained in our multilayer and time-varying networks into simpler summary statistics. A simple yet useful measure in this case is what we coin as *edge presence*, which is the fraction of time an edge is present (that is, $A_{ij}^{b}\left(t\right)=1$ ) during one full EEG sleep session. Formally,

$$P_{ij}^{b}={\left\langle A_{ij}^{b}\left(t\right)\right\rangle }_{t}$$ (5)

**FIGURE 3:**
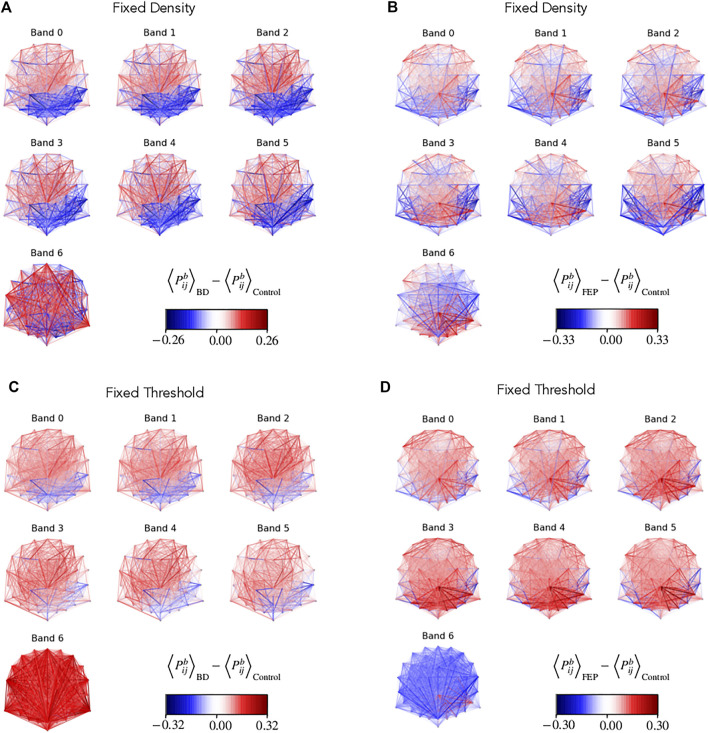
Network edge presence highlights differences between groups. Multilayer EEG fixed-density **(A, B)** or fixed-threshold **(C, D)** networks, with edges colored according to the average BD **(A, D)** or FEP **(C, D)** presence minus the corresponding average value of control patients. Edge presence is a measure of the fraction of time an edge is active, see Methods for details. The four panels use a divergent colormap that is blue for negatives values, red for positive values, and becomes gradually transparent as values approach zero. Overall, the panel visually shows clear differences between BD and control patients, and between FEP and control patients.

Figure 3 shows the group differences of $P_{ij}^{b}$ for each edge (*i*, *j*) and each band when comparing BD patients with controls (panels A, C), as well as FEP patients with controls (panels B, D). This analysis is shown both for fixed-density networks (A, B) and for fixed-threshold networks (C, D). In both cases we see differences in the parieto-occipital area, but the signal is stronger for fixed-density networks. If we focus on Figure 3A, for instance, we see that BD patients tend to have a lower edge presence in the parieto-occipital area (strong blue edges). Notice that we employ a colorbar that goes from red to transparent to blue, so that edges that do not have strong differences are effectively not drawn. Overall, the figure shows important differences in the parieto-occipital area, with a similar but stronger signal for fixed-density networks.

### 2.4 Parieto-Occipital Correlations and Clustering Measures Differ Between Groups

Motivated by the results shown visually in Figure 3, we construct a parieto-occipital (PO) specific measure. Selecting the 18 electrodes of that region (see methods for details), we compute the difference of PO presence between the PO area and the rest.

$$P_{\text{PO}}={\left\langle P_{ij}^{b}\right\rangle }_{\left(ij\right)\in PO}-{\left\langle P_{ij}^{b}\right\rangle }_{\left(ij\right)\notin PO}$$ (6)

Additionally, we also consider the average clustering coefficient, the average shortest path length and the betweenness centrality as measures related to clustering and information navigability as candidates to better quantify the differences that we see in Figure 3.

**FIGURE 4:**
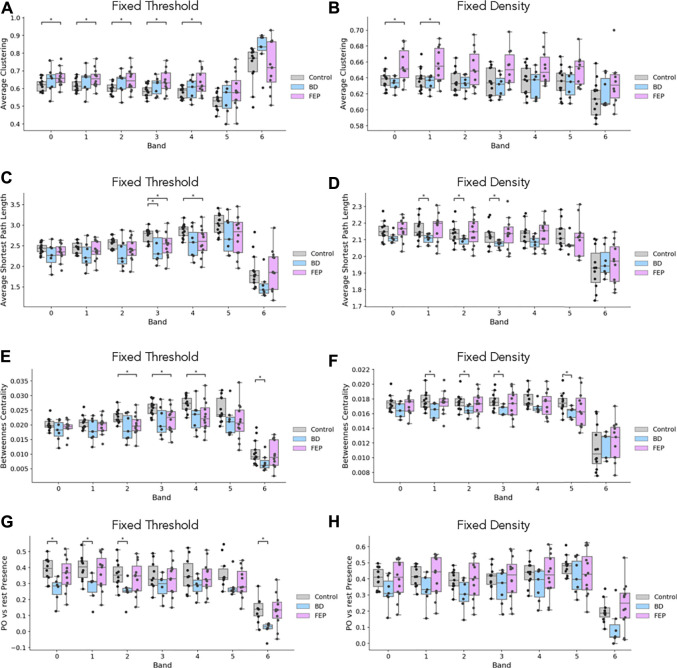
EEG network measures evidence differences between groups. Boxplots of average clustering **(A, B)**, average shortest path length **(C, D)**, betweenness centrality **(E, F)** and parieto-occipital presence **(G, H)** for control (gray), BD (blue) and FEP (pink) patients. Panels in the left column correspond to fixed-threshold networks, while panels in the right column correspond to fixed-denstiy networks.

Figure 4 shows boxplots of these four measures comparing, BD and FEP patients with control subjects. Statistically significant differences are marked with a star, see Methods for details. Panels (A, B) show that for bands 1 to 4 (that is, between 1 and 16 Hz), FEP patients have a higher clustering coefficient when compared to controls, while panels (C, D) shows some significant results on the same range of frequencies for the average shortest path length, both for BD and for FEP patients. Turning to betweenness centrality, panel E shows that when using fixed-threshold networks, FEP patients significantly differ from controls in bands 2, 3, and 4 (2–16 Hz) Interestingly, when looking at the parieto-occipital relative presence (panels G, H), we observe a different pattern of marked differences between BD and control patients for lower frequency bands, 0.5–4 Hz. This is consistent with the fact that the more standard network measures used in panels A to F treat all nodes under the same footing, independently of the brain region they correspond to, while PO presence is a tailor-made measure, specifically designed to capture the visual results of Figure 3 taking into account the location of parieto-occipital electrodes.

### 2.5 Network Measures Correlate With Edge Density

**FIGURE 5:**
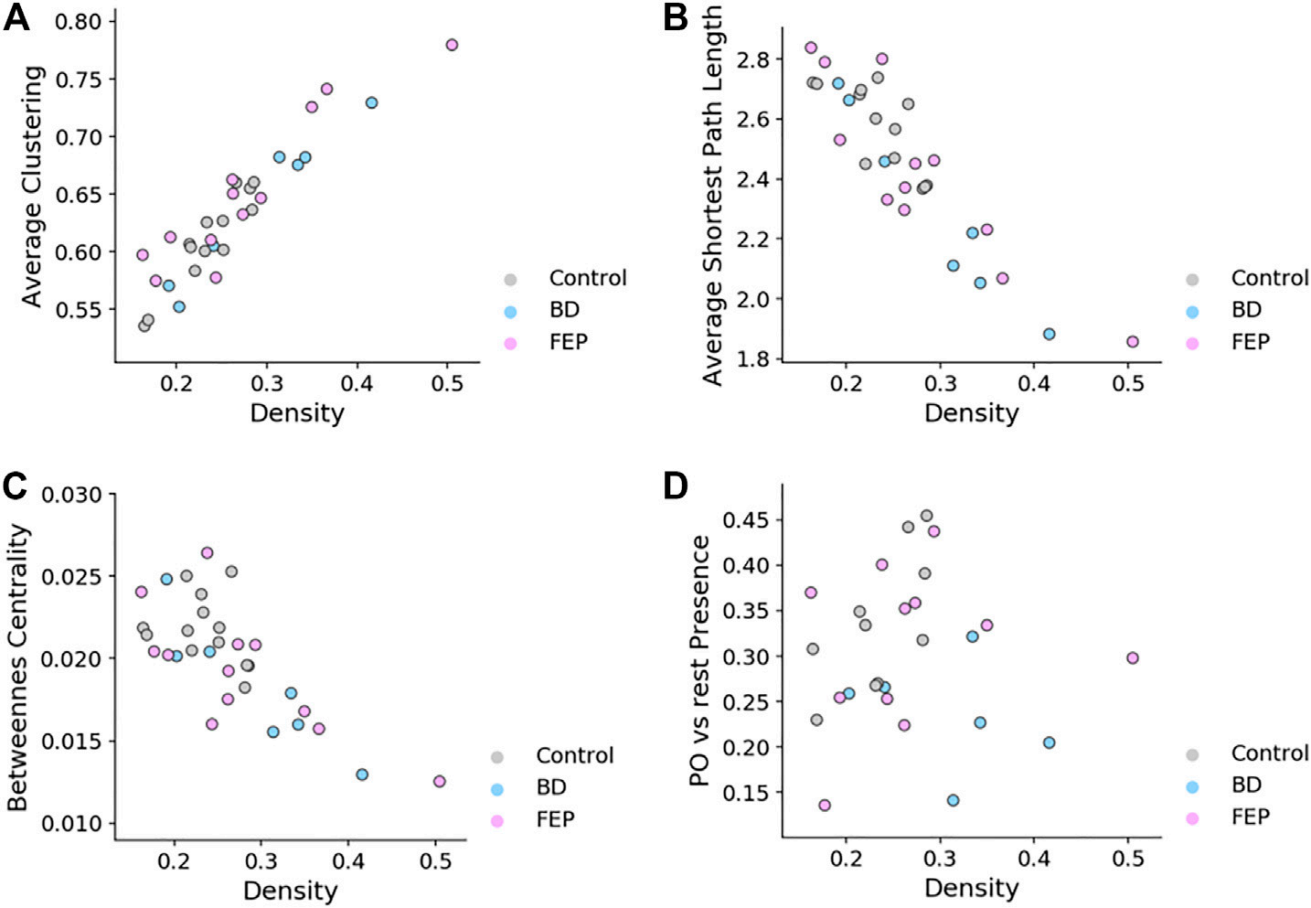
Fixed-threshold networks yield measures that correlate with edge density. Scatter plots of average clustering **(A)**, average shortest path length **(B)**, betweenness centrality **(C)** and parieto-occipital presence **(D)** vs. network density, for fixed-threshold networks. Overall, the panel shows that all measures except parieto-occipital presence correlate with network density.

It is interesting to ask if the network measures shown in Figure 4 are correlated with network edge density, for the case of fixed-threshold networks. Figure 5 shows how indeed edge density is a strong driver of average clustering coefficient, average shortest path length and betweenness centrality for all patient groups, but not of parieto-occipital presence. This is consistent with the fact that, by construction, *P*_PO_ is a relative difference of two averages taken on the same network.

## 3 Discussion

Hd-EEG represents an attractive method to study brain function by providing non-invasive spatio-temporal measurements of brain activity with possible applications to disease diagnosis and monitoring. While it is relatively easy to obtain large amount of data from individual subjects, extracting useful information from hd-EEG recordings is a challenging task. Hd-EEG only provides an indirect far-field measurement of the underlying electrical activity and is intrinsically subject to noise. Furthermore, hd-EEG recordings typically involve noisy signals recorded in parallel through different electrodes for long time periods so that even the mere visualization of the data is complex.

Network representations have been shown in the past to provide a useful tool to highlight the connectivity and spatio-temporal correlation of brain activity as revealed from EEG or other measurments such as fMRI. Due to the complexity of hd-EEG recordings, multilayer networks are more appropriate to represent the data since they provide separate visualization for potentially crucial features of EEG signals such as the frequency band and/or the time dependence. An effective network representation of hd-EEG recordings should be able to extract most of the relevant information from the signal cross-correlation. To address this issue, we use the IJSD to quantify information content in the multilayer network (Domenico and Biamonte, 2016) and adjust correlation threshold parameters to maximize it. In this way, we obtain a multilayer networks that maximizes the information content of the underlying hd-EEG recordings and test it on a set of EEG data obtained from patients with mental health issue, as well as healthy control subjects.

Statistical analysis on the resulting multilayer networks reveals a number of distinguishing topological features between patients and the control group. In particular, observed differences in parieto-occipital edge presence appear to be particularly relevant. These results indicate a stronger correlation of EEG signals in that area for BD patients with respect to control subjects, a feature that warrants further study and could potentially be used as a diagnostic tool.

An important issue in our analysis is that most statistical indicators crucially depend on the density of edges present in the network. To discount this effect, we constructed and analyzed constant-density multilayer networks. While our analysis only considers pairwise correlations, future work could also extend our analysis to the study of interactions between groups of nodes (Battiston et al., 2020).

We applied our strategy to a particular set of EEG recordings from patients with mental disorders, but the methodology could readily be generalized and applied to a variety of pathological conditions. It would be interesting for instance to use our multilayer network approach to predict the response of individual patients to specific drugs. Finally, the analysis of EEG signals could be enriched by measuring at the same time other physiological signals, such as heartbeat or respiration adding further layers to the network, in the spirit of the emerging field of network physiology (Bashan et al., 2012; Bartsch et al., 2015; Ivanov et al., 2016).

## 4 Methods

### 4.1 Data

Hd-EEG recordings where obtained from San Paolo Hospital in Milano. In particular, the dataset consists of sleep EEG recordings from 12 FEP patients (Eight males and four females, mean age 21.0 ± 3.77), seven BD patients (Three males and four females, mean age 34.57 ± 7.09), and 13 control subjects (Six males and seven females, mean age 25.61 ± 10.64). All participants underwent an in-laboratory sleep hd-EEG recording with a 64-electrode Easycap net designed to enhance electrode contact with the scalp (BrainAmp, Brain Products GmbH, Gilching, Germany). The night of the recording, all subjects were accommodated in a sleep suite and allowed to sleep within 1 h of their usual bedtime. All subjects were recorded throughout the night and until they woke up naturally the next morning. Table 1 shows the average length of recording sessions and total sleep time for each participant group. The headset has 64 unipolar electrodes positioned following the standard 10–20 system, and include two channels that record eye movements (one for vertical movements and one for horizontal movements). All recordings had a sampling frequency of 500 Hz. Data was provided in anonymized form as pairs of *.set* and *.fdt* files.

**TABLE 1:** Recording time and sleep time. Sleep time is obtained by visual scoring according to the American Academy of Sleep Medicine (AASM) Manual for the Scoring of Sleep and Associated Events (Berry, R. B., Brooks, R., Gamaldo, C. E. and Susan, M. 2012). All values expressed in minutes.

	FEP	BD	Healthy control
Recording Time (mean ± S.D.)	431.02 ± 136.94	526.06 ± 44.84	489.22 ± 42.44
Total Sleep Time (mean ± S.D.)	300.02 ± 115.75	351.77 ± 102.38	361.47 ± 73.92

### 4.2 Data Preprocessing

Our preprocessing pipeline transforms the raw EEG recordings into correlation tensors of the form $C_{ij}^{b}\left(t\right)$ , with (*i*, *j*) denoting and edge between electrodes *i* and *j*, *b* a specific frequency band, and *t* a 30-s epoch. The steps we carry are as follows:

1. Epochs division: divide the raw signal into epochs of approximately 30 s, see below for details, obtaining a signal *S*_*i*_(*t*) for electrode *i* and epoch *t*.
2. Artifact correction: apply eye-movement correction.
3. Bands division: divide the corrected signal into seven frequency bands. This gives a signal $S_{i}^{b}\left(t\right)$ with *b* ∈ {0, *…*, 6}.
4. Correlation analysis: compute electrode-to-electrode Pearson correlations, obtaining a correlation tensor of the form $C_{ij}^{b}\left(t\right)$ .

Epochs division: We divide EEG recordings into epochs of around 30 s following Aboalayon et al. (2016). To be precise, each epoch has a length of 2^14^ raw time points which, at a sampling frequency of 500 Hz, corresponds to 32.768 s. This choice is particularly convenient because pure powers of two allow for faster discrete Fourier transform calculations.

Artifact correction: Following Gratton et al. (1983), we correct for eye-movements using a linear regression equation of the form

Y=XB (7)

where *Y* corresponds to the EEG data (62 channels in our case), *X* corresponds to the eye-movement data (Two channels in our case), and *B* is the regression coefficient matrix to be determined. Solving for *B* *via* least squares, the corrected signal X* is computed as

$$X^{*}={\left(X-YB\right)}^{T}$$ (8)

Bands division: We use seven frequency bands, numbered from 0 to 6 throughout the manuscript, which logarithmically interpolate the 0.5–64 Hz range typical of brain waves.

• Band 0: (0.5, 1) Hz.
• Band 1: (1, 2) Hz.
• Band 2: (2, 4) Hz.
• Band 3: (4, 8) Hz.
• Band 4: (8, 16) Hz.
• Band 5: (16, 32) Hz.
• Band 6: (32, 64) Hz.

### 4.3 Correlation Analysis

We use the Pearson correlation coefficient to measure the strength and direction of dependence between the signals *x*_*i*_, *x*_*j*_ recorded by two electrodes *i*, *j*,

$$C_{ij}=\frac{cov\left(x_{i},x_{j}\right)}{\sigma_{x_{i}}\sigma_{x_{j}}}.$$ (9)

Repeating this measurement for each band *b* and timepoint *t*, we get a full correlation tensor $C_{ij}^{b}\left(t\right)$ .

### 4.4 Jensen-Shannon Divergence

We use Jensen-Shannon Divergence (JSD) as a distance measure between networks, in the framework of spectral entropies (Domenico and Biamonte, 2016). For a pair of networks with density matrices *ρ* and *σ*, the JSD is defined as

$$J\left(\rho \|\sigma \right)=S\left(\frac{\rho +\sigma }{2}\right)-\frac{1}{2}\left[S\left(\rho \right)+S\left(\sigma \right)\right],$$ (10)

where *S*(*ρ*) is the spectral entropy of the network,

$$S\left(\rho \right)=\log_{2}Z+\frac{\tau }{\ln2}\mathrm{Tr}\left[L\rho \right],$$ (11)

with *L* denoting the Laplacian, *τ* diffusion time and the density matrix *ρ* defined as

$$\rho =\frac{e^{-\tau L}}{Z},\;Z=\mathrm{Tr}\left(e^{-\tau L}\right)$$ (12)

### 4.5 Network Measures

Parieto-occipital edge presence: The parieto-occipital area is mapped to the following electrodes: P7, P5, P3, P1, PZ, P2, P4, P6, P8, PO7, PO3, PO4, PO8, O1, OZ, O2, and IZ. From this list, the parieto-occipital presence is computed as explained in the main text, mainly the difference of average presence between nodes in the parieto-occipital area and the rest.

Clustering Coefficient: We use the standard definition of clustering coefficient,

$$c_{i}=\frac{2\cdot t_{i}}{k_{i}\cdot \left(k_{i}-1\right)}$$ (13)

as implemented in the networkx python library (Hagberg et al., 2008), where *t*_*i*_ is the number of triangles in which node *i* is involved and *k*_*i*_ is the node degree. Averaging over all nodes, we define the clustering coefficient of the network as

$$c=\frac{1}{N}\overset{N}{\underset{i=1}{\sum }}c_{i}$$ (14)

Betweenness Centrality: We use the convention of Brandes (2008), which defines a node-dependent quantity as follows:

$$c_{B}\left(i\right)\;=\;\frac{2}{\left(N-1\right)\left(N-2\right)}\underset{j,k\in V}{\sum }\frac{\sigma \left(j,k|i\right)}{\sigma \left(j,k\right)}$$ (15)

where *σ*( *j*, *k*|*i*) is the number of shortest path that connect nodes j and k that passes through i and *σ*( *j*, *k*|*i*) = 0 if *i* = *j*, *k*. *σ*( *j*, *k*) is the total number of shortest path connecting j and k and *σ*( *j*, *k*) = 1 if *j* = *k*. By convention the fraction $\frac{\sigma \left(j,k|i\right)}{\sigma \left(j,k\right)}$ is considered zero if both elements are zero. We then average over all nodes to get a single measure for each network:

$$BC\;=\;\frac{1}{N}\overset{N}{\underset{i=1}{\sum }}c_{B}\left(i\right).$$ (16)

Average Shortest Path Length: We start from the standard definition of average shortest path length (ASPL) for a connected graph *G*

$$a_{G}\;=\;\frac{1}{N\cdot \left(N-1\right)}\underset{i\neq j}{\sum }d\left(i,j\right).$$ (17)

where *d*(*i*, *j*) is defined as the length of the shortest path connecting two nodes, *j*. If *i* and *j* belong to two different connected components *d*(*i*, *j*) is said to be infinite, while *d*(*i*, *j*) = 0 if *i* = *j*.

In our setting, networks can have more than one connected component, and we do not want to limit ourselves to the largest connected component as important information could be missed. Hence we employ a weighted version of the ASPL,

$$wa_{G}\;=\;\frac{\sum_{c=1}^{n_{c}}a_{c}\cdot w_{c}}{\sum_{c=1}^{n_{c}}w_{c}}$$ (18)

where *n*_*c*_ is the number of connected components with more than two nodes and *w*_*c*_ = *N*_*c*_ ⋅ (*N*_*c*_ − 1), *N*_*c*_ is the number of nodes of component *c*. This formulation takes into account the ASPL of all nodes but effectively gives more weight to the larger components.

### 4.6 Statistical Analysis

Group differences are assessed with a two-sided *T*-test without assuming equal variances between groups, as implemented in the *ttest_ ind* function from the *scipy* Python scientific library. Cases marked as significant (∗) in Figure 4 correspond to a *p*-value below 0.05.

### 4.7 Ethical Approval

Data from the SPINDLE-1 study, approved by the Milan Area A Interhospital Ethics Committee (Approval n. 22864). All participants signed an informed consent for participation in the SPINDLE-1 study.

## Data Availability

The raw data supporting the conclusions of this article will be made available by the authors, without undue reservation.
